# Antiviral Action of Tryptanthrin Isolated from *Strobilanthes cusia* Leaf against Human Coronavirus NL63

**DOI:** 10.3390/biom10030366

**Published:** 2020-02-27

**Authors:** Yu-Chi Tsai, Chia-Lin Lee, Hung-Rong Yen, Young-Sheng Chang, Yu-Ping Lin, Su-Hua Huang, Cheng-Wen Lin

**Affiliations:** 1 PhD Program for Health Science and Industry, College of Health Care, China Medical University, Taichung 40402, Taiwan; chijoan0805@gmail.com; 2 Graduate Institute of Biomedical Sciences, College of Medicine, China Medical University, Taichung 40402, Taiwan; a0989016192@gmail.com; 3 Department of Medical Laboratory Science and Biotechnology, China Medical University, Taichung 40402, Taiwan; c20234@hotmail.com; 4 Department of Cosmeceutics, China Medical University, Taichung 40402, Taiwan; chlilee@mail.cmu.edu.tw; 5 Chinese Medicine Research and Development Center, China Medical University Hospital, Taichung 40447, Taiwan; 6 Chinese Medicine Research Center, China Medical University, Taichung 40402, Taiwan; hungrongyen@gmail.com; 7 Graduate Institute of Chinese Medicine, School of Chinese Medicine, College of Chinese Medicine, China Medical University, Taichung 40402, Taiwan; 8 Department of Chinese Medicine, China Medical University Hospital, Taichung 40447, Taiwan; 9 Research Center for Traditional Chinese Medicine, Department of Medical Research, China Medical University Hospital, Taichung 40447, Taiwan; 10 Department of Biotechnology, Asia University, Wufeng, Taichung 41354, Taiwan; shhuang@asia.edu.tw

**Keywords:** *Strobilanthes cusia*, tryptanthrin, indigodole B, human coronavirus NL63, antiviral, virucidal

## Abstract

*Strobilanthes cusia* (Nees) Kuntze is a Chinese herbal medicine used in the treatment of respiratory virus infections. The methanol extract of *S. cusia* leaf contains chemical components such as β-sitosterol, indirubin, tryptanthrin, betulin, indigodole A, and indigodole B that have diverse biological activities. However, the antiviral action of *S. cusia* leaf and its components against human coronavirus remains to be elucidated. Human coronavirus NL63 infection is frequent among immunocompromised individuals, young children, and in the elderly. This study investigated the anti-Human coronavirus NL63 (HCoV-NL63) activity of the methanol extract of *S. cusia* leaf and its major components. The methanol extract of *S. cusia* leaf effectively inhibited the cytopathic effect (CPE) and virus yield (IC_50_ = 0.64 μg/mL) in HCoV-NL63-infected cells. Moreover, this extract potently inhibited the HCoV-NL63 infection in a concentration-dependent manner. Among the six components identified in the methanol extract of *S. cusia* leaf, tryptanthrin and indigodole B (5a*R*-ethyltryptanthrin) exhibited potent antiviral activity in reducing the CPE and progeny virus production. The IC_50_ values against virus yield were 1.52 μM and 2.60 μM for tryptanthrin and indigodole B, respectively. Different modes of time-of-addition/removal assay indicated that tryptanthrin prevented the early and late stages of HCoV-NL63 replication, particularly by blocking viral RNA genome synthesis and papain-like protease 2 activity. Notably, tryptanthrin (IC_50_ = 0.06 μM) and indigodole B (IC_50_ = 2.09 μM) exhibited strong virucidal activity as well. This study identified tryptanthrin as the key active component of *S. cusia* leaf methanol extract that acted against HCoV-NL63 in a cell-type independent manner. The results specify that tryptanthrin possesses antiviral potential against HCoV-NL63 infection.

## 1. Introduction

*Strobilanthes cusia* (Nees) Kuntze is a member of the family *Acanthaceae*, widely distributed in northeast India, Bangladesh, southern China, the Himalayan region, Myanmar, and Taiwan [1]. *S. cusia* root and leaf extracts have been used extensively as a traditional herbal medicine with anti-inflammatory, antipyretic, antimicrobial, and antiviral activities [2,3]. The *S. cusia* root (named “Nan-Ban-Lan-Gen” in Chinese) has been commonly used to treat infections by respiratory virus, such as influenza viruses, mumps virus, and severe acute respiratory syndrome (SARS) coronavirus [4,5]. Several bioactive components from the *S. cusia* root, including strobilanthes A, 3H-benzoxazolinone, and aurantiamide acetate, have exhibited antiviral activity against influenza A and hepatitis B virus infections [5,6]. The *S. cusia* leaf (called “Da-Ching-Yeh” in Chinese) is generally used for the production of indigo dyes (Indigo Natruralis, named “Qing Dai” in Chinese), displaying antibacterial, anti-inflammatory, and antipyretic properties [7,8]. *S. cusia* leaves contain effective chemical components with antibacterial, anti-inflammatory and antitumor activities, including β-sitosterol, indirubin, tryptanthrin (6,12-dihydro-6,12-dioxoindolo-(2,1-b)-quinazoline), betulin, indigodole A, indigodole B (5a*R*-ethyltryptanthrin), strobilanthosides A–C, and phenylethanoidglycosides [9,10]. Notably, indirubin and its derivatives modulate influenza A virus-induced inflammation and have been suggested as antiviral and immunomodulatory agents against influenza A virus infection [3,11,12,13,14]. β-sitosterol exerts an inhibitory effect on the in vitro enzymatic activity of SARS coronavirus 3C-like protease [15]. However, the antiviral capability of *S. cusia* leaf extract is yet to be elucidated; clarifying its properties will prove to be relevant to respiratory virus infections.

Human coronavirus NL63 (HCoV-NL63) belongs to the family *Coronaviridae*, as denoted shortly after the emergence of severe acute respiratory syndrome coronavirus (SARS-CoV) [16,17]. HCoV-NL63 is an enveloped virus with a single-strand, positive-sense RNA genome nearly 26–32 kb in size [18]. The spike protein, the major envelope protein of HCoV-NL63, specifically binds to the zinc peptidase angiotensin-converting enzyme 2 (ACE2) identified as the receptor for pathogenic coronaviruses SARS-CoV and COVID-19 (formerly known as Wuhan coronavirus, 2019-nCoV, and SARS-CoV-2) [16,17,18,19]. The virus is prevalent during spring and winter seasons in temperate zone countries. HCoV-NL63 is primarily associated with immunocompromised patients with respiratory illnesses, young children, and the elderly [20]. Symptoms of HCoV-NL63 infection include common cold, rhinorrhea, cough, fever, tachypnea, and obstructive laryngitis [21]. Recently, HCoV-NL63 induced an outbreak of severe respiratory disease in long-term care institutions in Louisiana. In 20 cases with patients aged between 66 and 96, six patients with pneumonia were hospitalized and three patients died [22]. Moreover, HCoV-NL63 was isolated from the blood, urine, and stool samples of children with febrile illness, causing systemic illness in rural Haiti [23]. HCoV-NL63 has become one of the primary pathogens in respiratory viral diseases; however, there are no effective antivirals for treating HCoV-NL63 infection. 

This study explored the anti-HCoV-NL63 activity exhibited by the methanol extract of *S. cusia* leaf and its major chemical components, including β-sitosterol, indirubin, tryptanthrin, betulin, indigodole A, and indigodole B, by means of cytopathic effect (CPE), virus yield, infectivity, time-of-addition/removal, and virucidal activity assays. 

## 2. Materials and Methods

### 2.1. Cell and Virus

HCoV-NL63 provided by Dr. Lia van der Hoek at the Department of Medical Microbiology, University of Amsterdam, was used in the antiviral assays [20]. Rhesus monkey kidney epithelial cells (LLC-MK2) were cultured in Modified Eagle’s Medium (HyClone) supplemented with 100 U/mL penicillin-streptomycin, 100 mM nonessential amino acids (Corning), 100 mM sodium pyruvate, and 10% fetal bovine serum (Gibco). LLC-MK2 cells were used to amplify the titer of HCoV-NL63 for the antiviral assay. Human airway Calu-3 cells were also used to test the antiviral activity of indicated components and were cultured in MEM supplemented 10% FBS. 

### 2.2. Preparation of S. cusia Leaf Methanol Extract and Its Related Compounds

The powder from *S. cusia* leaf collected in Putian City, Fujian Province, China was subjected to treatment in a GMP pharmaceutical factory in China controlled by Sheng Chang Pharmaceutical Co., Ltd. in Zhongli District, Taoyuan City, Taiwan. The powder of *S. cusia* leaf (Lot. No. BR0308980) was purchased and further credited at the Chinese Medicine Research and Development Center, China Medical University Hospital, Taiwan, as described in a previous report [9]. The extract of *S. cusia* leaf powder (10 kg) was generated four times by methanol extraction (36 L each) at room temperature. The chemical components β-sitosterol, indirubin, tryptanthrin, botulin, indigodole A, and 5a*R*-ethyltryptanthrin (indigodole B) were separated by partition experiments with EtOAc-H_2_O, hexane–90% MeOH(aq), and n-BuOH–H_2_O, purified using silica gel chromatography and identified by NMR spectroscropy, as described in a previous report [9].

### 2.3. MTT Cytotoxicity Test

Cytotoxicity of *S. cusia* extract and its identified compounds against LLC-MK2 and Calu-3 cells was evaluated by the MTT (3-(4,5-dimethylthiazol-2-yl)-2,5-diphenyltetrazolium bromide) assay. A total of 5 × 10^3^ cells per well were seeded overnight in a 96-well plate, and treated with 0, 5, 10, 50, 100, and 500 μg/mL of *S. cusia* extract or with 0, 0.4, 4, 40, and 400 μM of the indicated chemical components. After 48 h of treatment, 10 μL of MTT solution (5 mg/mL) in phosphate-buffered saline (PBS) was added to each well and incubated for 4 h in the incubator at 37 °C and 5% CO_2_. Lastly, 100 μL isopropanol was added into each well to dissolve the formazan crystals in cells. The OD_570-630_ of each well was measured using a micro-ELISA reader; cell viability was calculated as the ratio of OD_570-630_ of treated cells to OD_570-630_ of mock cells.

### 2.4. Cytopathic Effect Reduction and Virus Yield Inhibition Assays

In the CPE reduction assay, 2 × 10^5^ LLC-MK2 cells per well were grown overnight in 6-well plates, infected with HCoV-NL63 at 0.01 multiplicity of infection (MOI), and immediately treated with the indicated concentrations of *S. cusia* leaf extract and the purified compounds (β-sitosterol, indirubin, tryptanthrin, betulin, indigodole A, and indigodole B). Images of CPE in infected cells were captured using a microscope_._ After 24, 36 and 48 h of incubation at 37 °C and 5% CO_2_, HCoV-NL63-induced CPEs such as cell swelling, rounding, vacuoles, and eventual detachment were imaged using a microscope, in which vacuoles in infected cells were predominant [24]. In addition, the effect of stomach acid on inactivation of the tryptanthrin was further assayed. Tryptanthrin was incubated in 0.01 N HCl (pH 2.0) for 15 or 60 min, neutralized with PBS, and the residual activity of CPE reduction in HCoV-NL63 infected cells at the MOI of 0.01 was evaluated. In the virus yield reduction assay, the supernatant from untreated or treated infected cells was diluted to quantify the virus titer using the plaque assay. Serial dilutions of the supernatant were added onto the monolayer of LLC-MK2 cells for 1 h incubation at 37 °C to enable attachment and entry; the cells were overlaid using 2% agarose in MEM containing 2% FBS. After 48 h of incubation, the cell monolayer was stained with naphthol blue-black dye; the plaques in the LLC-MK2 cell monolayer were counted to determine the virus titer. The virus yield reduction was calculated as the reduced rate of plaque content in the treated infected cells compared to the untreated infected cells. Fifty percent (50%) inhibitory concentration (IC_50_) was calculated as the percentage of virus yield reduction by the abovementioned concentrations of the extract and active compounds. In addition, the antiviral activity of the active compounds was examined in human lung epithelial Calu-3 cells at 32 °C; 1 × 10^5^ Calu-3 cells per well were cultured overnight in 6-well plates, infected with HCoV-NL63 at an MOI of 0.05, and immediately treated with the indicated concentration of the active compounds. CPE reduction and infectivity inhibition by the active compounds were enabled and further evaluated 36 h post-infection_._

### 2.5. Infectivity Inhibition Assay Using Immunofluorescent Staining

To measure the inhibitory activity of the extract and active compounds on the replication of HCoV-NL63, LLC-MK2 and Calu-3 cells were infected by HCoV-NL63, simultaneously treated with the extract and the active compounds, and then subjected to the assay according to the methods of the CPE reduction assay, as described above. Later, the cells were fixed with 4% paraformaldehyde in PBS for 30 min, quenched with 50 mM NH_4_CI for 15 min, and permeabilized and blocked using 1% albumin bovine (Affymetrix) plus triton X-100 (Thermo Fisher, Waltham, MA, USA). After a 4 h incubation at 4 °C, the cells were reacted overnight with the 1/2000 dilution of HCoV-NL63-immunized sera in 1% BSA at 4 °C, and treated with 1/3000 dilution of Alexa Fluor anti-mouse IgG antibodies in 1% BSA for 1 h at 4 °C (Thermo Fisher). The cells were further stained with 4′,6-diamidino-2-phenylindole (DAPI) for 20 min at room temperature (Thermo Fisher). The image analysis of stained cells was recorded using fluorescent microscopy and the ImageJ2 software. The infectivity was represented as the ratio of HCoV-NL63-positive cells (red fluorescent signals) to total cells (blue fluorescent signals).

### 2.6. Analyzing the Inhibitory Effect on the Early and Late Stages Using the Time-of-Addition/RemovalAssay

The time-of-addition/removal assays included co-treatment/removal and post-infection treatment/removal modes. In the co-treatment/removal mode, the cells were infected by HCoV-NL63 (MOI = 0.01) and concurrently treated with the active compounds (0.4, 4, 40 μM). After 2 h of incubation, the mixture of virus and compound was removed; the infectivity inhibition assay was performed subsequently using immunofluorescent staining. In the post-infection treatment/removal mode, the cells were subjected to HCoV-NL63 infection for 2 h, treated with active compounds for an additional 2 h, and washed with PBS for removing the mixture of virus and the compound. The residual infectivity in both modes was determined by the relative levels of HCoV-NL63-positive cells. Moreover, the supernatant and the lysate from the infected cells in both modes were collected for examining the titers of extracellular virions using the plaque assay, as described above. In addition, HCoV-NL63 RNA genomes in LLC-MK2 cells treated by both modes were quantified using quantitative reverse transcription polymerase chain reaction (RT-qPCR). The total RNA from treated/infected cells at 24 h post-infection was extracted using the PureLink Mini Total RNA Purification Kit (ThermoFisher), reverse transcribed with the SuperScript III reverse transcriptase and oligo (dT), and analyzed using SYBR Green-based real time PCR with a HCoV-NL63-specific primer pair (5′-CACAGATTTTTGGACGGTTGC-3′ and 5′-TGTGTTGATAATAAATGGGGAGTG-3′), and a β-actin gene-specific primer pair (5′-CAACTGGGACGACATGGAGAAAAT-3′ and 5′-CCAGAGGCGTACAGGGATAGCAC-3′). The threshold cycle value (C_T_) of HCoV-NL63 RNA genomes and β-actin mRNAs in each sample was evaluated using 7300 Realtime PCR system (Applied Biosystems, Foster, CA, USA). The relative levels of HCoV-NL63 RNA genomes were normalized by β-actin mRNAs and calculated.

### 2.7. Construction, Expression, Purification, and In Vitro Trans-Cleavage Activity of Recombinant HCoV-NL63 Papain-Like Protease 2 (PLP2)

The papain-like protease 2 (PLP2) gene encoded in nucleotides 5018-5932 of the human coronavirus NL63 genome (GenBank Accession No. JX504050) was amplified using reverse-transcriptase PCR with a specific primer pair (5′-GGCGCGGATCCAAGAATGATAATGTAGTT-3′ and 5′-GATCCAAGCTTACTAACAATTGTTGGAAC-3′). The forward and reverse primers contained a BamH1 and HindIII restriction site, respectively. The PLP2 gene was cloned into the pET24a vector (Novagen, Darmstadt, Germany) and expressed in *Escherichia coli* strain BL21(DE3) after induction by 4 mM isopropyl-β-d-thiogalactopyranoside (IPTG) for 4 h at 37 °C. Recombinant HCoV-NL63 PLP2 was purified with the HisTrap Kit (Amersham, Piscataway, NJ, USA) using the supernatant from bacterial cells sonicated in 10 mM imidazole buffer after centrifugation (10,000 × *g* for 20 min). Meanwhile, the concentration, purity, and integrity of recombinant HCoV-NL63 PLP2 protein were determined using the Bio-Rad protein assay reagent, SDS-PAGE, and Western blotting. The sample of the purified PLP2 protein was dissolved in loading buffer without 2-mercaptoethanol and boiled for 10 min, resolved in a 10% SDS-PAGE gel, and stained with Coomassie Brilliant Blue (Sigma, Darmstadt, Germany). The PLP2 protein separated by SDS-PAGE gel was electrophoretically transferred to a nitrocellulose paper that was blocked with 5% skimmed milk and then reacted with the appropriately diluted anti-His Tag monoclonal antibody and goat anti-mouse IgG antibodies conjugated with alkaline phosphatase in a 2 h incubation. After washing thrice with 1% TBS containing 0.05% Tween 20 (TBST), the blots were developed with TNBT/BCIP (Gibco). Furthermore, in vitro *trans*-cleavage assay using horseradish peroxidase (HRP) containing the LXGG motif as a substrate was used to analyze the protease activity of recombinant HCoV-NL63 PLP2. Indicated concentrations of recombinant HCoV-NL63 PLP2 were reacted with 100 µl of substrate reagent containing 0.5 µg HRP in PBS in the 96-well plate. After 2 h incubation at 37 °C, the residual HRP activity was measured by the chromogen solution containing 2,2′-azino-di-3-ethylbenzthiazoline-6-sulfonate and hydrogen peroxide. Relative *trans*-cleavage activity of recombinant HCoV-NL63 PLP2 was calculated as 1−(OD_405 with PLP2_)/(OD_405 without PLP2_). In addition, the tryptanthrin-mediated inhibition of the protease activity of recombinant HCoV-NL63 PLP2 was subsequently quantified by the in vitro *trans*-cleavage assay with the addition of tryptanthrin at different concentrations. The relative inhibitory activity of tryptanthrin on HCoV-NL63 PLP2 was calculated as

1−(OD405_without PLP2_−OD405_with PLP2 and tryptanthrin_)/(OD405_without PLP2_−OD405_with PLP2_).

### 2.8. Assays on Virucidal Activity

To assess whether the active compound directly affects viral infectivity (virucidal activity), 10^5^ pfu HCoV-NL63 were incubated with the indicated concentrations of the active compounds for 1 h at 37 °C. For reducing the cell-based antiviral effects exerted by the active compounds, 100 μL of the 1000-fold dilution of the virus/compound mixture was added into the LLC-MK2 cells monolayer in 6-well plates for analyzing the residual infectivity using the plaque assay, as described above. The virucidal activity was evaluated according to the reduction in the percentage of infectious virions after the direct reaction of HCoV-NL63 with the active compounds compared to that in the untreated group. Furthermore, after 1 h incubation at 37 °C, 15 μL of each virus/compound mixture was directly spotted onto a nitrocellulose membrane for the dot-blot assay with anti-HCoV-NL63 antibodies for evaluating the integrity of HCoV-NL63 treated with tryptanthrin. The nitrocellulose membrane was blocked with 5% skim milk in Tris-Buffered Saline plus 0.1% Tween-20 (TBST) for 2 h at 4 °C, incubated overnight with anti-HCoV-NL63 antibodies, and reacted with HRP-conjugated anti-mouse IgG antibodies (Invitrogen, Carlsbad, CA, USA). After washing with TBST, immunoreactive spots of HCoV-NL63 structural proteins (spike and envelope proteins) were developed with ECLTM Western Blotting Detection Reagents (GE Healthcare, Chicago, IL, USA), and imaged by the Multi-function Gel Image System (MultiGel-21) (Gentaur, San Jose, CA, USA).

### 2.9. Statistical Analysis

One-way ANOVA followed by Scheffe’s post-hoc test using SPSS 12.0 (SPSS, Inc., Armonk, NY, USA) was used to analyze data from three independent experiments. A *p* value < 0.05 indicated statistically significant results.

## 3. Results

### 3.1. Antiviral Activity of S. cusia Leaf Methanol Extract against HCoV-NL63

**Figure 1:**
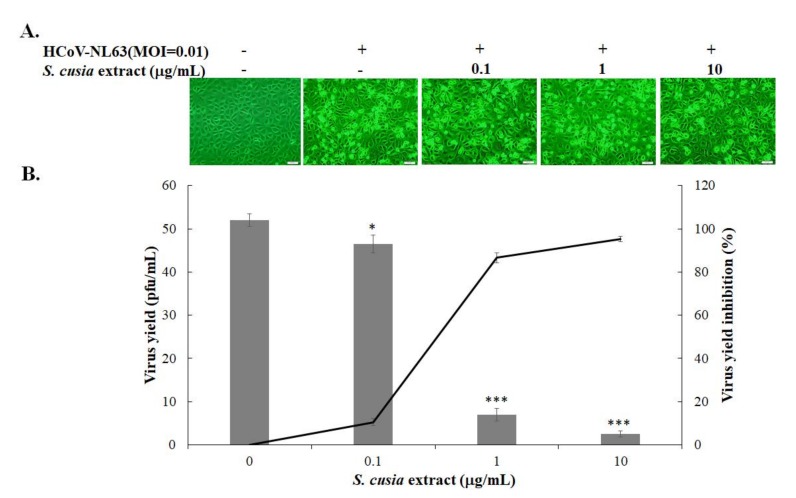
Inhibitory effects of *S. cusia* leaf methanol extract on viral cytopathicity and progeny virus production in HCoV-NL63-infected cells. LLC-MK-2 cells were infected with HCoV-NL63 at 0.01 multiplicity of infection (MOI) and simultaneously treated with the extract. Virus-induced cytopathic effects were imaged 36 h post-infection (hpi) by microscopy (**A**). The supernatant from treated infected cells was harvested 36 hpi and serially diluted for determining the HCoV-NL63 yield in the supernatant using the plaque assay (**B**, right y axis). The rate of virus yield inhibition was calculated based on the ratio of the loss in the titer of the treated group to mock-treated group (**B**, left y axis). +, add; −, not add; *, *p* value < 0.05; ***, *p* value < 0.001 compared with mock-treated infected cells. Scale bar, 100 μM.

**Figure 2:**
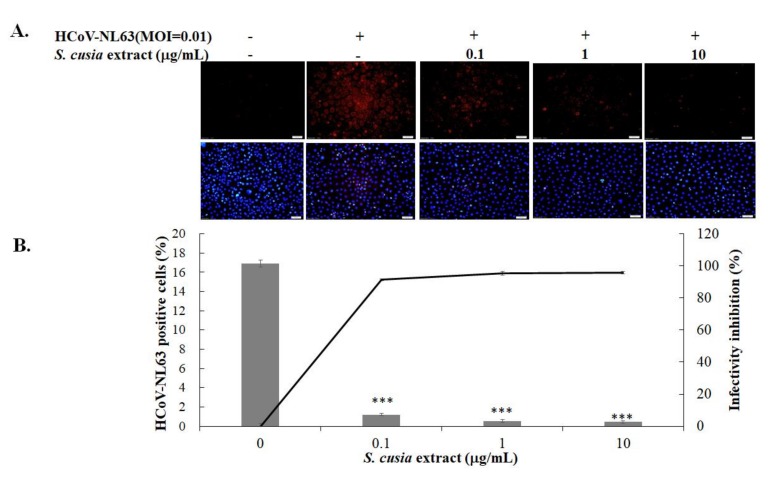
Inhibition of HCoV-NL63 infectivity by *S. cusia* leaf methanol extract. LLC-MK2 cells were infected with HCoV-NL63 and immediately treated with the extract for 36 h at 37 °C, and then subjected to immunofluorescence staining with anti-HCoV-NL63 immunized sera and secondary antibody Alexa Fluor anti-mouse IgG (**A**, top); total cells were stained with 4′,6-diamidino-2-phenylindole (DAPI) (**A**, bottom). Infectivity inhibition was determined according to the decrease in the ratio of HCoV-NL63-positive cells to total cells (**B**). +, add; −, not add; ***, *p* value < 0.001 compared with untreated infected cells. Scale bar, 100 μM.

The methanol extract of the *S. cusia* leaf was less cytotoxic to LCC-MK2 cells (CC_50_ > 100 μg/mL) (Figure S1A, Table 1). Anti-HCoV-NL63 activity of *S. cusia* leaf extract at the concentrations of 0.1, 1, and 10 μg/mL was further evaluated using the CPE reduction and virus yield inhibition assays (Figure 1). HCoV-NL63 induced an observable vacuolation in LLC-MK2 cells at 37 °C 48 h post-infection, as described in the prior report [24]. The *S. cusia* leaf methanol extract reduced HCoV-NL63-induced CPE in LLC-MK2 cells in a concentration-dependent manner (Figure 1A). The plaque assay indicated that the *S. cusia* leaf methanol extract exhibited a strong inhibitory effect against the production of progeny HCoV-NL63 virions released into the cultured media of LLC-MK2 cells at 37 °C 48 h post-infection (Figure 1B). The IC_50_ value of *S. cusia* leaf methanol extract against virus yield in LLC-MK2 cells was 0.64 μg/mL (Figure 1B, Table 1). Immunofluorescent staining with anti-HCoV-NL63-immunized sera revealed that the *S. cusia* leaf methanol extract inhibited the HCoV-NL63-infected LLC-MK2 positivity at 37 °C 36 h post-infection in a concentration-dependent manner as well (Figure 2A). Image analysis of HCoV-NL63-positivity ratio in total cells from each well indicated that 0.1 μg/mL of *S. cusia* leaf methanol extract reduced HCoV-NL63 infectivity by more than 90% (the ratio of HCoV-NL63-positive cells) (Figure 2B). The results revealed that the *S. cusia* leaf methanol extract exhibited a potent antiviral activity against HCoV-NL63.

### 3.2. Action of Active Components from S. cusia Leaf Extract against HCoV-NL63

**Figure 3:**
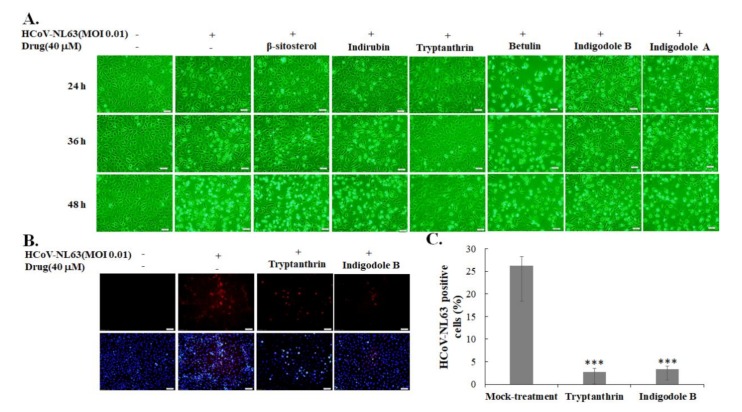
Inhibitory effects of *S. cusia* extract components on viral cytopathicity and HCoV-NL63 infectivity. LLC-MK2 cells were infected with HCoV-NL63 and immediately treated with 40 μM of the indicated components for 36 h at 37 °C, photographed for evaluating the relative cytopathic effect (CPE) levels (**A**), and the residual HCoV-NL63 infectivity was determined using immunofluorescence staining with anti-HCoV-NL63 antibodies (**B**, top) and DAPI (**B**, bottom). The ratio of HCoV-NL63-positive cells was calculated as the percentage of positive cells to total cells (**C**). +, add; −, not add; ***, *p* value < 0.001 compared with mock-treatment group. Scale bar, 100 μM.

**Figure 4:**
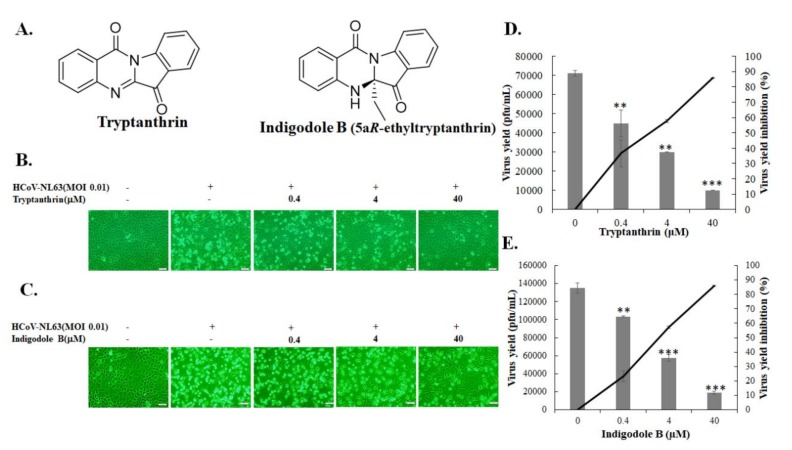
Inhibition of viral cytopathicity and virus yield by tryptanthrin and indigodole B. The structure of tryptanthrin and indigodole B is shown in (**A**). Images of CPE reduction by tryptanthrin and indigodole B were captured 36 hpi (**B**,**C**). The inhibitory activity of tryptanthrin and indigodole B on virus yield was calculated based on the ratio of titer loss in the treated group to that in the mock-treated group (**D**,**E**). +, add; −, not add; **, *p* value < 0.01; ***, *p* value < 0.001 compared with untreated infected cells. Scale bar, 100 μM.

The chemical components of the *S. cusia* leaf methanol extract included β-sitosterol, indirubin, tryptanthrin, betulin, indigodole A, and indigodole B, as described in our prior report [9]. These chemical components (at 40 μM concentration) were studied for the antiviral activity exhibited against HCoV-NL63 using the CPE reduction assay (Figure 3). Among them, tryptanthrin at 40 μM exhibited the strongest antiviral activity with a significant reduction in HCoV-NL63-induced CPE in LLC-MK2 cells 36 and 48 h post-infection (Figure 3A). Indigodole B (5a*R*-ethyltryptanthrin) at 40 μM concentration was ranked the second highest in terms of anti-HCoV-NL63 activity in reducing virus-induced CPE 36 and 48 h post-infection (Figure 3A). In addition, tryptanthrin and indigodole B, which exhibit a low cytotoxicity in LLC-MK2 cells (CC_50_ < 400 μM), significantly reduced the HCoV-NL63 infectivity, as detected by immunofluorescent staining assay (Figure 3B–C, Figure S1B–C, Table 1). Notably, indigodole B is a derivative of tryptanthrin conjugated with the ethyl group at C-5a in *R* configuration, and is also known as 5a*R*-ethyltryptanthrin (Figure 4A). Subsequently, the antiviral activity of 0.4, 4, and 40 µM tryptanthrin and indigodole B was determined using CPE inhibition and virus yield reduction assays (Figure 4B–E). In the CPE reduction assay, tryptanthrin at 0.4, 4, and 40 µM significantly reduced virus-induced CPE in LLC-MK2 cells at 37 °C 48 h post-infection, whereas the same effect was noted only for 40 μM indigodole B (Figure 4B–C). Notably, tryptanthrin resisted stomach juice (0.01 N HCl, pH 2.0) activity for 15 and 60 min, during which time it induced CPE reduction and infectivity inhibition in HCoV-NL63 infected cells as well (Figures S2 and S3, Table 1). Moreover, the plaque assay revealed that tryptanthrin and indigodole B exerted a potent inhibitory effect on the in vitro progeny production of HCoV-NL63 (Figure 4D–E), wherein IC_50_ values for virus yield were 1.52 μM and 2.60 μM for tryptanthrin and indigole B, respectively (Table 1). Therefore, similar to the *S. cusia* leaf methanol extract, tryptanthrin and indigodole B (5a*R*-ethyltryptanthrin) exhibited low cytotoxicity and high antiviral potential against HCoV-NL63.

### 3.3. Evaluation of Anti-HCoV-NL63 Activity of Tryptanthrin in Human Lung Epithelial Cells

**Figure 5:**
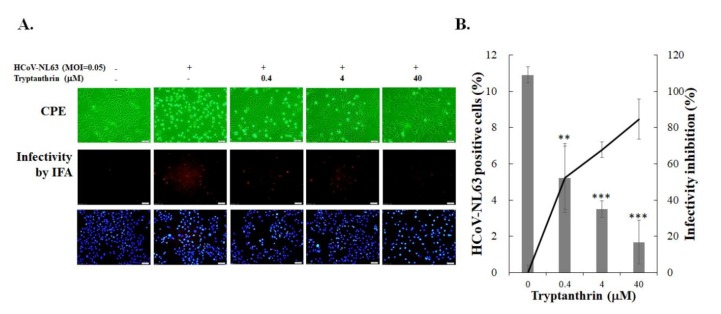
Tryptanthrin inhibited HCoV-NL63 infectivity in human airway epithelial cells. Calu-3 cells were infected with HCoV-NL63 and immediately treated with tryptanthrin for 36 h at 32 °C. Images of relative CPE levels in each group were captured (**A**, top). In addition, the cells were subjected to immunofluorescence staining with anti-HCoV-NL63 antibodies plus secondary antibody Alexa Fluor anti-mouse IgG (**A**, middle) and DAPI (**A**, bottom). Infectivity inhibition activity was determined based on the change in the percentage of HCoV-NL63-positive cells (**B**). +, add; −, not add; **, *p* value < 0.01; ***, *p* value < 0.001 compared with untreated infected group. Scale bar, 100 μM.

Tryptanthrin was less toxic to human lung epithelial Calu-3 cells (CC_50_ = 173.2 μM) than indigodole B (CC_50_ = 72.5 μM) (Figure S4, Table 1). To examine the antiviral efficacy of the highest active component, tryptanthrin, in LLC-MK2 cells, the infectivity inhibitory effect of tryptanthrin against HCoV-NL63 was tested in Calu-3 cells using CPE reduction and infectivity inhibition assays (Figure 5). The antiviral assays with human lung epithelial Calu-3 cells were performed at 32 °C and analyzed 36 h post-infection (Figure 5). Tryptanthrin inhibited the HCoV-NL63-induced CPE in a concentration-dependent manner (Figure 5A, top). Immunofluorescent staining indicated that tryptanthrin significantly reduced the percentage of HCoV-NL63-positive cells (Figure 5A, middle and bottom), markedly reducing the HCoV-NL63 infectivity with an IC_50_ value of 0.30 μM in human lung epithelial Calu-3 cells (Figure 5B, Table 1). The results confirmed that tryptanthrin exhibited a cell-type independent anti-HCoV-NL63 activity with IC_50_ values of 1.52 μM and 0.30 μM in LCC-MK2 and Calu-3 cells, respectively.

### 3.4. Antiviral Mechanism Underlying Tryptanthrin Action against HCoV-NL63

**Figure 6:**
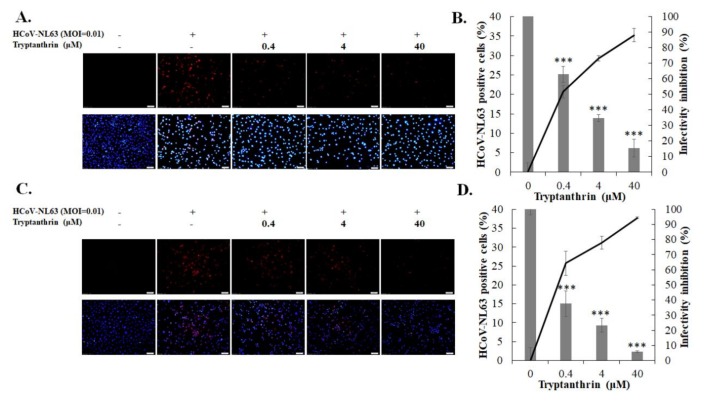
Time-of-addition and removal assay for analyzing antiviral action of tryptanthrin against HCoV-NL63. The cell monolayer was infected with HCoV-NL63 and treated with tryptanthrin simultaneously (**A**, early stage), or 1 hpi (**C**, late stage). After a 2 h of incubation, the virus/tryptanthrin mixture was removed; the cell monolayer was washed with PBS and cultured for an additional 36 h with incubation at 37 °*C*, and then subjected to immunofluorescence staining using anti-HCoV-NL63 antibodies plus secondary antibody Alexa Fluor anti-mouse IgG (**A**,**C**, top) and DAPI (**A**,**C**, bottom). Infectivity was determined according to the percentage of HCoV-NL63-positive cells (**B**,**D**). +, add;−, not add; ***, *p* value < 0.001 compared with untreated infected group. Scale bar, 100 μM.

**Figure 7:**
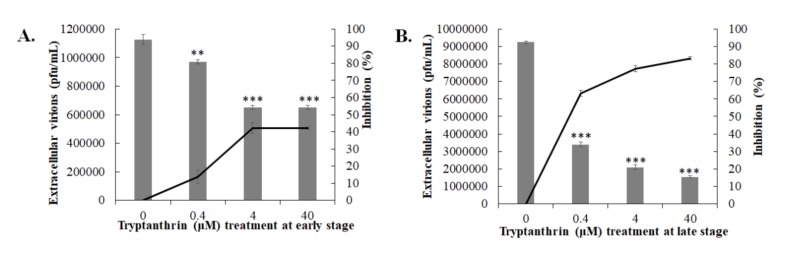
Time-of-addition and removal assay for examining the effect of tryptanthrin on the early and late stages of HCoV-NL63 replication. The cell monolayer was infected with HCoV-NL63 and treated with tryptanthrin simultaneously (**A**, early stage), or 1 hpi (**B**, late stage). After 2 h of incubation, the virus/tryptanthrin mixture was removed; the cell monolayer was washed with PBS and cultured for an additional 36 h with incubation at 37 °C. The extracellular virus yield in the supernatant was determined using the plaque assay; the inhibition rate was analyzed based on the loss in the ratio of virus titer in treated group to that in the mock-treated group. **, *p* value < 0.01; ***, *p* value < 0.001 compared with mock-treated infected cells.

To explore whether tryptanthrin interferes with HCoV-NL63 replication, different modes of time-of-addition/removal assay were performed to examine the inhibitory effect of tryptanthrin on early and late stages of replication (Figure 6 and Figure 7). The infectivity assay with immunofluorescent staining indicated that tryptanthrin directly reduced the percentage of HCoV-NL63-positive cells in both modes (Figure 6A,C), exhibiting a strong inhibitory effect in the early and late stages of HCoV-NL63 replication. Tryptanthrin was more potent in impeding the late stage (IC_50_ = 0.06 μM) than the early stage (IC_50_ = 0.32 μM) (Figure 6D vs. Figure 6B, Table 1). In the plaque assay, tryptanthrin markedly reduced the production of extracellular virions in these two modes, implying that it significantly blocked virus production at the early and late stages (Figure 7). In addition, tryptanthrin exhibited a higher inhibitory activity against the production of extracellular virions in the late stage (IC_50_ = 0.05 μM) than in the early stage (IC_50_ = 6.99 μM) (Figure 7A,B, Table 1).

### 3.5. Tryptanthrin-Mediated Inhibition of HCoV-NL63 RNA Genome Synthesis and Papain-Like Protease 2 Activity

**Figure 8:**
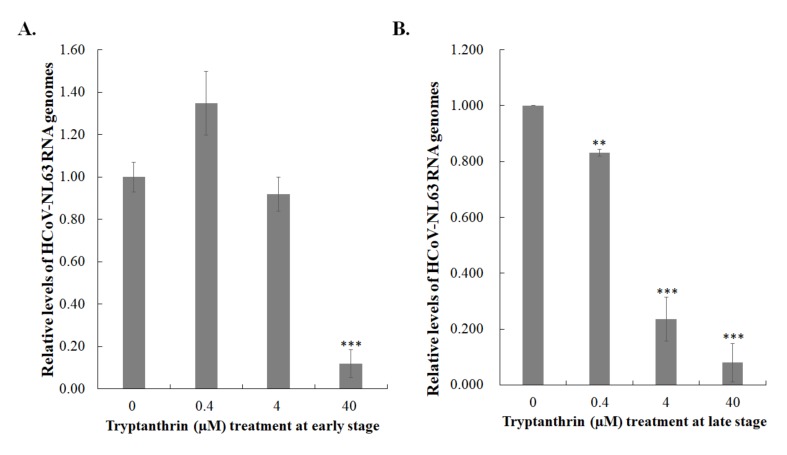
Inhibitory effect of tryptanthrin on the synthesis of viral RNA genome in the early and late stages of HCoV-NL63 replication. The cell monolayer was infected with HCoV-NL63 and treated with tryptanthrin simultaneously (**A**, early stage), or 1 hpi (**B**, late stage). After 2 h of incubation, the virus/tryptanthrin mixture was removed; the cell monolayer was washed with PBS and cultured for an additional 24 h with incubation at 37 *°*C. Total RNA from treated and transfected cells was extracted and reverse transcribed with HCoV-NL63-specific primers. Relative viral RNA genomes were measured by quantitative PCR and normalized by β-actin mRNA. **, *p* value < 0.01; ***, *p* value < 0.001 compared with mock-treated infected cells.

**Figure 9:**
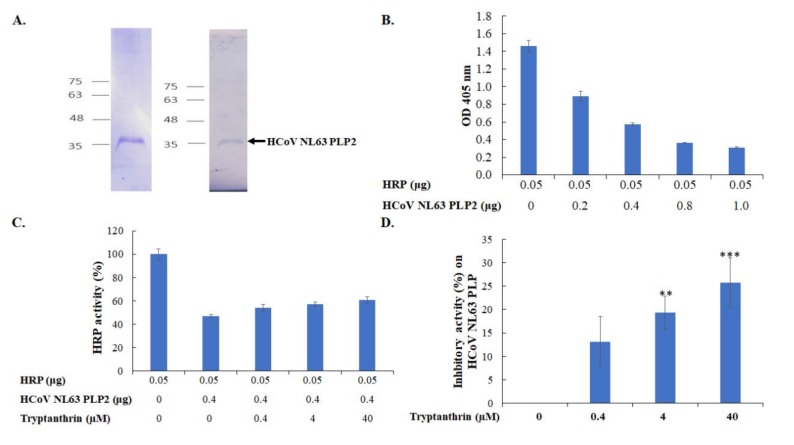
Inhibition of in vitro *trans*-cleavage activity of recombinant HCoV-NL63 papain-like protease 2 by tryptanthrin. The purified recombinant HCoV-NL63 papain-like protease 2 (PLP2) was analyzed by 10% SDS-PAGE with Coomassie blue staining (**A**, right) and Western Blotting with anti-His tag antibodies (**A**, left). The *trans*-cleavage activity of HCoV-NL63 PLP2 was evaluated using the ELISA with horseradish peroxidase (HRP) containing an LXGG motif as the substrate. After 2 h of incubation at 37 °C, the enzyme activity of the residual HRP (in intact form) was detected using a chromogen reagent (ABTS/H_2_O_2_). The ELISA product was measured at A_405 nm_ (**B**). Tryptanthrin at indicated concentrations was added into the mixture of HCoV-NL63 PLP2 and HRP, and then incubated for 2 h at 37 °C. Lastly, the enzyme activity of the residual HRP (in intact form) was determined using the chromogen reagent (ABTS/H_2_O_2_), calculated as 1−(OD405 with PLP2)/(OD405 without PLP2) (**C**). Moreover, the relative inhibition of the cleavage activity of PLP2 by tryptanthrin was estimated as 1−(OD405 without PLP2−OD405 with PLP2 plus tryptanthrin)/(OD405 without PLP2−OD405 with PLP2) (**D**). **, *p* value < 0.01; ***, *p* value < 0.001 compared with the mock-treated group.

Tryptanthrin exhibited a higher effective inhibitory effect during the late stage of HCoV-NL63 replication cycle than the early stage; thus, the functional activity of possible targets, such as viral RNA polymerase and protease, was further measured in the time-of-addition/removal assay and the in vitro *trans*-cleavage test. The RT-qPCR assay revealed that tryptanthrin reduced the number of HCoV-NL63 RNA genomes in the post-infection treatment/removal mode more effectively than in the co-treatment/removal mode (Figure 8). The result indicated that tryptanthrin possibly reduced the transcriptional action of HCoV-NL63 RNA-dependent RNA polymerase during viral replication. Moreover, the inhibitory effect of tryptanthrin on the protease activity of HCoV-NL63 PLP2 was further detected using the in vitro *trans*-cleavage assay (Figure 9). Recombinant HCoV-NL63 PLP2 protein with a His tag was synthesized in *E. coli* and purified using the immobilized metal affinity chromatography. SDS-PAGE and Western blotting assays determined the purity and integrity of recombinant HCoV-NL63 PLP2 protein with a molecular weight of approximately 36 kDa (Figure 9A). Recombinant PLP2 protein exhibited proteolytic activity on the substrate HRP in a concentration-dependent manner (Figure 9B). Notably, tryptanthrin exhibited dose-dependent inhibition of recombinant PLP2-mediated *trans*-cleavage on HRP (Figure 9C,D). The results indicated that the antiviral activity of tryptanthrin against HCoV-NL63 was associated with the inhibitory action on viral RNA genome synthesis and PLP2 activity during the later stage of in vitro replication that was linked with the viral infectivity and virion production.

### 3.6. Virucidal Activity of Tryptanthrin against HCoV-NL63

**Figure 10:**
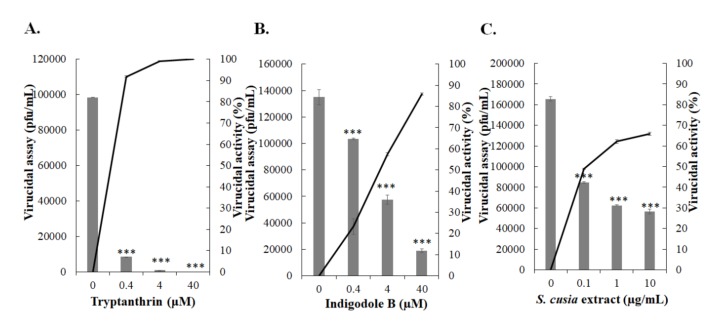
Virucidal activity of tryptanthrin, indigodole B, and *S. cusia* extract. Tryptanthrin (**A**), indigodole B (**B**), or the extract (**C**) at the indicated concentrations was mixed with HCoV-NL63 (10^5^ pfu), and incubated at 37 *°*C for 1 h. The 1000-fold dilution of the compound/virus mixture was added into the LLC*-*MK2 cell monolayer in 6-well plates for examining the residual infectivity by plaque assay. Virucidal activity was calculated based on the percentage of residual plaques in each treated group. ***, *p* value < 0.001 compared with untreated infected cells.

**Figure 11:**
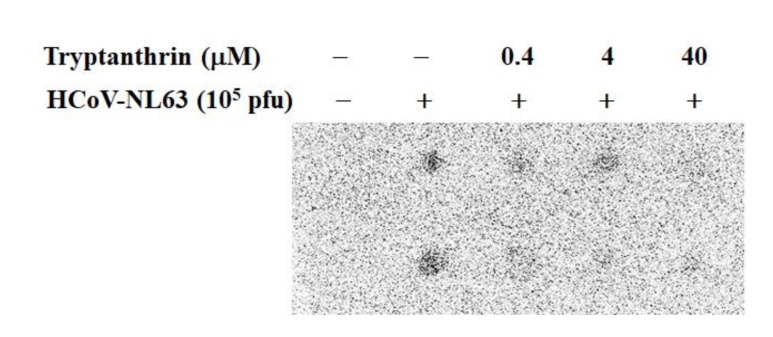
Dot-blotting assay of the HCoV-NL63/tryptanthrin mixture incubated at 37 *°*C for 1 h. Tryptanthrin at indicated concentrations was mixed with HCoV-NL63 (10^5^ pfu), and then incubated *at 37 °*C for 1 h. Fifteen microliters of the compound/virus mixture was added onto the nitrocellulose membrane. The nitrocellulose membrane was blocked with 5% skim milk in Tris-Buffered Saline plus 0.1% Tween-20 (TBST) for 2 h, incubated with anti-HCoV-NL63 antibodies overnight, and then reacted with HRP-conjugated anti-mouse IgG antibodies. After washing with TBST, immunoreactive spots of HCoV-NL63 were developed with ECLTM Western Blotting Detection Reagents, and then imaged by the Multi-function Gel Image System. +, add; −, not add;

To determine the virucidal activity of tryptanthrin against HCoV-NL63, the virucidal assay was performed by pre-incubation of the high-titer HCoV-NL63 with tryptanthrin at 37 °C for 1 h (Figure 10A). For determining the residual infectivity, the 1000-fold dilution of the mixture was used in the plaque assay based on the negligible effects of tryptanthrin on plaque formation. Tryptanthrin exhibited a significant virucidal activity in a concentration-dependent manner at 37 °C*,* and the IC_50_ value of tryptanthrin for virucidal activity was 0.06 μM (Figure 10A, Table 1). Subsequently, the virucidal activity of indigodole B and *S. cusia* leaf methanol extract were evaluated (Figure 10B,C). Notably, indigodole B and *S. cusia* leaf methanol extract exhibited virucidal activity in a concentration-dependent manner for which the IC_50_ values were 2.09 μM for indigodole B and 0.12 μg/mL for *S. cusia* extract (Figure 10B,C, Table 1). The results indicated that tryptanthrin and indigodole B functioned as critical components eliciting virucidal activity in *S. cusia* leaf methanol extract. Moreover, the dot-blotting assay using the virus/compound mixture after a 1-h incubation at 37 °C, which was used to examine the effect of tryptanthrin on surface protein integrity of HCoV-NL63, revealed that tryptanthrin altered the antigenic structure of HCoV-NL63 structural proteins (spike and envelope proteins, which were associated with the inactivation of viral infectivity) (Figure 11).

## 4. Discussion

This study was the first to demonstrate the antiviral efficacy of *S. cusia* leaf methanol extract against HCoV-NL63 (Figure 1, Figure 2 and Table 1). The *S. cusia* extract significantly reduced progeny virus production (IC_50_ = 0.64 μg/mL) and inhibited viral infectivity (<0.01 μg/mL). Notably, tryptanthrin and indigodole B were originally identified as the compounds in the *S. cusia* leaf methanol extract with highest activity against HCoV-NL63 (Figure 3, Figure 4 and Figure 5, Table 1). Tryptanthrin was first reported as the distinct antiviral agent to reduce HCoV-NL63-induced CPE and to drastically inhibit the progeny virus production in a concentration-dependent manner (IC_50_ = 1.52 μM) (Figure 4). Indigodole B, recently identified in *S. cusia* leaf methanol extract [9], is a tryptanthrin derivative conjugated with the ethyl group at C-5a (5a*R*-ethyltryptanthrin), and exhibited intense antiviral activity with an IC_50_ of 2.60 μM against virus yield (Figure 4). Previous studies revealed several active antiviral compounds in the *S. cusia* extract against Herpes simplex virus-1 (IC_50_ = 49.3 μM by lupeol) [4], and influenza A virus (IC_50_ = 29.2 μM by strobilanthes A, IC_50_ = 46.0 μM by 2(3H)-benzoxazolinone, and IC_50_ = 31.48–71.57 μg/mL by aurantiamide acetate) [5,6]. Moreover, β-sitosterol was denoted as the inhibitor of the SARS coronavirus 3C-like protease [15], and exerted marginal inhibitory activity against HCoV-NL63-induced CPE. Therefore, *S. cusia* contains several active chemical constituents like tryptanthrin and indigodole B against various viruses, such as coronaviruses and influenza viruses.

The antiviral activity of tryptanthrin and indigodole B was first reported in this study (Figure 3, Figure 4, Figure 5, Figure 6, Figure 7, Figure 8 and Figure 9, Table 1). Tryptanthrin and indigodole B were less toxic to LCC-MK2 and Calu-3 cells (Table 1). Previous reports showed that tryptanthrin had no significant cytotoxic effect on the survival rates of human normal cells such as human foreskin fibroblasts and mast cells [25,26]. Tryptanthrin exhibited better antiviral activity than its derivative indigodole B (Figure 3 and Figure 4), exhibiting potent anti-HCoV-NL63 activity by virus yield reduction in LLC-MK2 cells (IC_50_ = 1.52 μM) and infectivity inhibition in Calu-3 cells (IC_50_ = 0.30 μM) (Figure 4 and Figure 5, Table 1). Therefore, tryptanthrin inhibited HCoV-NL63 infectivity in Calu-3 cells at 32 °C more effectively than that in LLC-MK2 cells at 37 °C (Figure 4 and Figure 5, Table 1). The results revealed that tryptanthrin functioned as a potent inhibitor with cell-type-independent antiviral activity against HCoV-NL63. Tryptanthrin significantly targeted viral enzymes like RNA-dependent RNA polymerase and PLP2 that are involved in the late stages of HCoV-NL63 replication, moderating the viral RNA genome synthesis and progeny virus production (Figure 6, Figure 7, Figure 8 and Figure 9). Tryptanthrin was first isolated from *S. cusia* and also extracted from the other indigoferous plants, *Persicaria tinctoria* and *Isatis tinctoria* (also known as *Isatis indigotica*) [27,28,29]. Tryptanthrin has a variety of pharmacological properties, such as antifungal (dermatophytes), antibacterial (*Helicobacter pylori*), anti-inflammatory, and antitumor (leukemia, breast and colon cancer cells) properties [30,31,32,33,34,35,36]. In addition, tryptanthrin displayed anti-angiogenic effect by improving skin lesions in psoriasis through regulation of ERK1/2 MAPK and PI3K-mediated expression of apelin [37]. Tryptanthrin exhibited anti-neuroinflammatory action by inactivating NF-κB pathway to suppress TLR4-MyD88-mediated inflammation [38]. Additionally, tryptanthrin exerted a hepatoprotective effect on oxidative stress through the activation of AMP-activated protein kinase and p38 mitogen-activated protein kinase in HepG2 cells [39]. Therefore, such pharmacological activities improved the antiviral potential of tryptanthrin against HCoV-NL63 and other viruses.

In addition to its antiviral activity, tryptanthrin demonstrated potent virucidal activity against HCoV-NL63 (IC_50_ *=* 0.06 μM) (Figure 10A, and Table 1). Additionally, indigodole B and the *S. cusia* leaf methanol extract exhibited effective virucidal activity against HCoV-NL63, with IC_50_ values of 2.09 μM for indigodole B and 0.12 μg/mL for *S. cusia* extract (Figure 10B,C, and Table 1). Therefore, tryptanthrin and indigodole B were suggested to be the key components in *S. cusia* leaf methanol extract that exerted the antiviral and virucidal effects against HCoV-NL63. Since tryptanthrin was one of the critical components in *Persicaria tinctoria* and *Isatis tinctoria* (Isatis indigotica), it might play a role in the antiviral properties of the extracts against Influenza A and B, dengue, Japanese encephalitis, and pseudorabies virus [11,40,41,42,43]. Notably, *Isatis tinctorial* extract exhibited virucidal activity against Japanese encephalitis virus and pseudorabies virus. However, tryptanthrin was not identified as the antiviral compound in the *Isatis tinctorial* extract, possibly owing to the difference in the separation methods. Therefore, more studies are necessary to elucidate the antiviral activity of tryptanthrin against Influenza A and B, dengue, Japanese encephalitis, and pseudorabies virus in future studies. 

## 5. Conclusions

This study indicates that tryptanthrin was identified as the major active component in *S. cusia* leaf methanol extract that inhibits HCoV-NL63 replication in a cell-type-independent manner. Interestingly, tryptanthrin serves a higher antiviral activity against HCoV-NL63 than indigodole B (5a*R*-ethyltryptanthrin) that has an additional ethyl moiety at C5a instead of double bond in tryptanthrin, revealing the double bond in quinazoline ring of tryptanthrin skeleton as the active contributor to prove the antiviral activity. Moreover, tryptanthrin specifically alters the antigenic structure of viral spike proteins and inhibits the cleavage activity of PLP2, as associated with virucidal activity and inhibiting the post-entry stage of HCoV-NL63 replication with IC_50_ of less than 0.1 μM. Interestingly, HCoV-NL63 spike protein shows a highly conserved sequence and structural similarity to SARS-CoV and COVID-19, linking it with the fact that all these viruses have the ACE2 receptor usage. Thus, tryptanthrin, exhibiting the virucidal action and impeding post-entry replication, might be developed as one of the first hit compounds against human coronaviruses.

## Figures and Tables

**Table 1:** Cytotoxicity and anti-human coronavirus NL63 (HCoV-NL63) activity of *Strobilanthes cusia* leaf methanol extract, indigodole B, and tryptantrin.

Tests	*S. cusia* Extract (μg/mL)^a^	Indigodole B (μM)^a^	Tryptanthrin (μM)^a^
**LCC-MK2 cells at 37 °C**			
Cytotoxicity (CC_50_)	> 100	> 4 00	> 400
Virus yield inhibition using plaque assay (IC_50_)	0.64 ± 0.43	2.60 ± 0.11	1.52 ± 0.13
Virucidal activity using plaque assay	0.12 ± 0.03	2.09 ± 0.89	0.06 ± 0.04
Infectivity inhibition with the compound in 0.01 N HCl (pH 2.0) for 15 min using IFA^b^ (IC_50_)			0.67 ± 0.03
Infectivity inhibition with the compound in 0.01 N HCl (pH 2.0) for 60 min using IFA (IC_50_)			1.58 ± 0.15
Infectivity inhibition at the early stage using IFA (IC_50_)			0.32 ± 0.05
Infectivity inhibition at the late stage using IFA (IC_50_)			0.06 ± 0.03
Production inhibition of extracellular virions at the early stage using plaque assay (IC_50_)			6.99 ± 2.18
Production inhibition of extracellular virions at the late stage using plaque assay (IC_50_)			0.05 ± 0.03
Calu-3 cells at 32 °C			
Cytotoxicity (CC_50_)		72.5 ± 0.77	173.2 ± 1.3
Infectivity inhibition using IFA (IC_50_)			0.30 ± 0.01

^a^ Mean ± standard deviation. ^b^ Immunofluorescence assay.

## Supplementary Materials

The following are available online at https://www.mdpi.com/2218-273X/10/3/366/s1.
