# Supplementary material for: Antiviral Action of Tryptanthrin Isolated from Strobilanthes cusia Leaf against Human Coronavirus NL63
Source: Biomolecules. 2020 Feb 27;10(3):366. doi: 10.3390/biom10030366 (PMC7175275; doi:10.3390/biom10030366)
Supplement: Supplementary file 1 [file biomolecules-10-00366-s001.pdf]

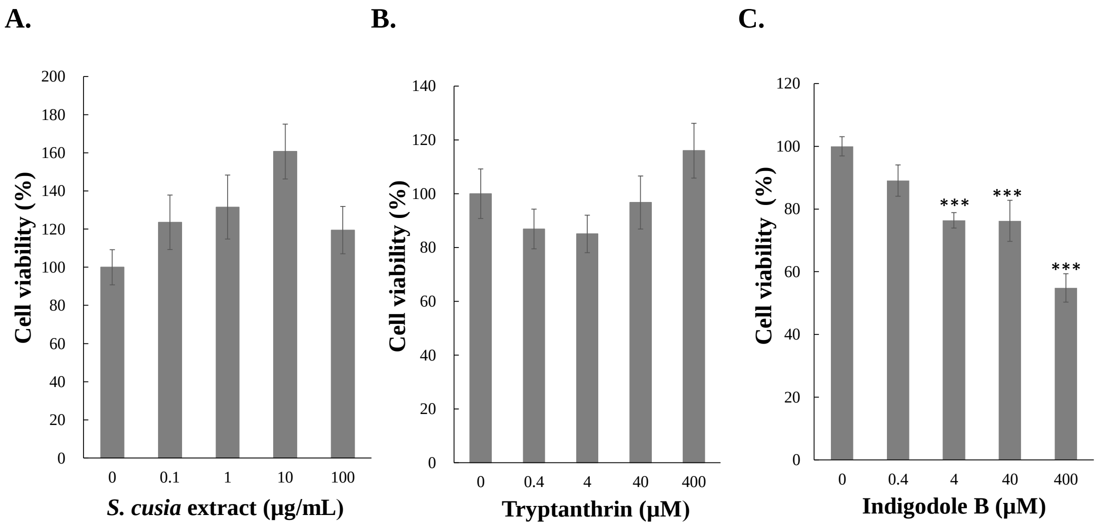

Supplemental Fig. 1. **Cell viability of LLC-MK2 cells treated with *S. cusia* leaf methanol extract (A), tryptanthrin (B), and indigodole B (5a*R*-ethyltryptanthrin) (C).** Cell viability of treated LLC-MK2 cells was evaluated using the MTT assay, and then calculated as the ratio of $OD_{570–630\ nm}$ of treated cells to $OD_{570–630\ nm}$ of untreated cells.

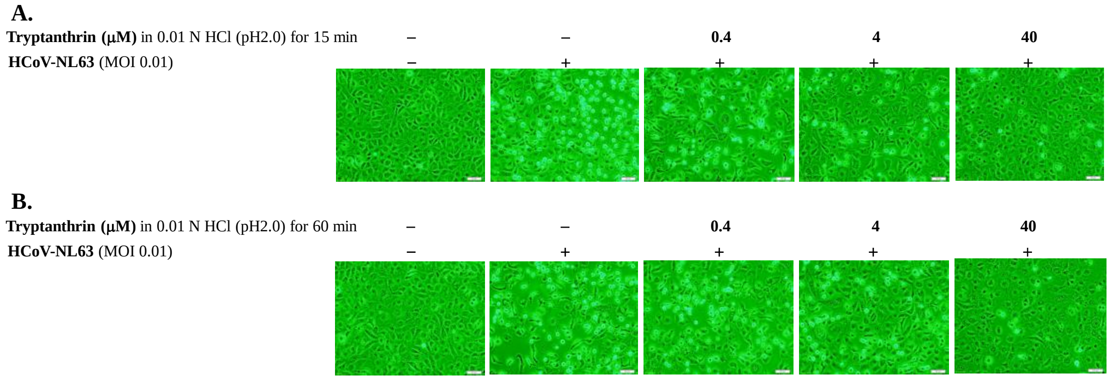

Supplemental Fig. 2. Inhibitory action of 0.01 N HCl-treated tryptanthrin on viral cytopathicity in HCoV-NL63 infected cells. LLC-MK-2 cells were infected with HCoV-NL63 at an MOI of 0.01 and simultaneously added along with the 0.01 N HCl-treated tryptanthrin for 15 (A) and 60 (B) min. Virus-induced cytopathic effect was imaged 36 h post-infection (hpi) by microscopy. Scale bar, 100 μm.

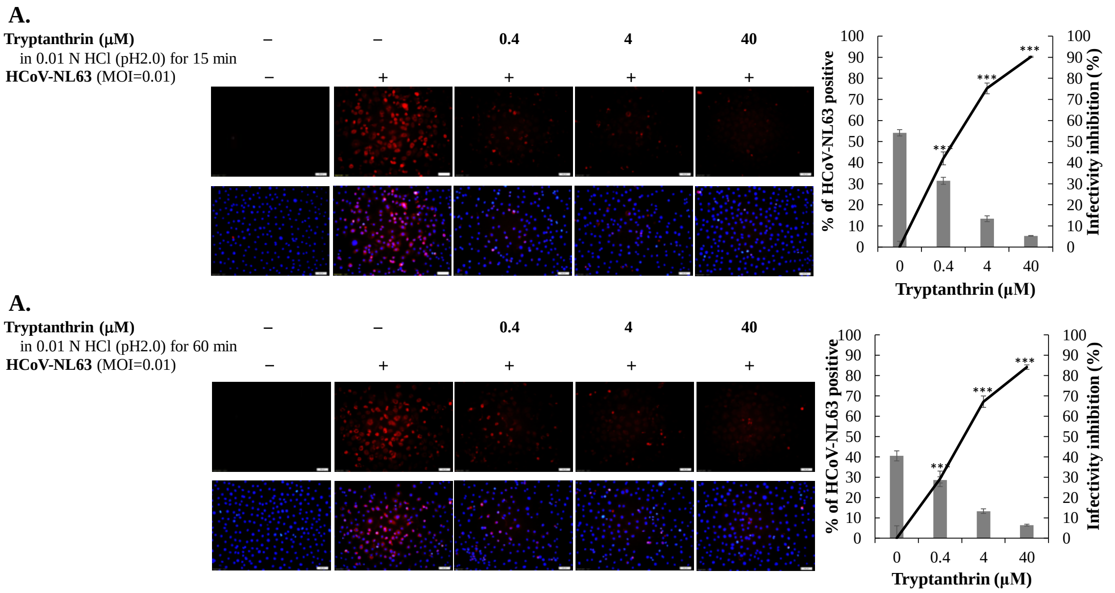

Supplemental Fig. 3. Inhibitory action of 0.01 N HCl-treated tryptanthrin on viral infectivity in HCoV-NL63 infected cells. LLC-MK-2 cells were infected with HCoV-NL63 at an MOI of 0.01 and simultaneously added along with the 0.01 N HCl-treated tryptanthrin for 15 (A) and 60 (B) min. After a 2 h of incubation, the virus/tryptanthrin mixture was removed; the cell monolayer was washed with PBS and cultured for an additional 36 h with incubation at 37 °*C*, and then subjected to immunofluorescence staining using anti-HCoV-NL63 antibodies plus secondary antibody Alexa Fluor anti-mouse IgG (A and B, the top left) and DAPI (A and B, the bottom left). Infectivity was determined according to the percentage of HCoV-NL63-positive cells (A and B, the right). ***, p value<0.001 compared with untreated infected group. Scale bar, 100 μm.

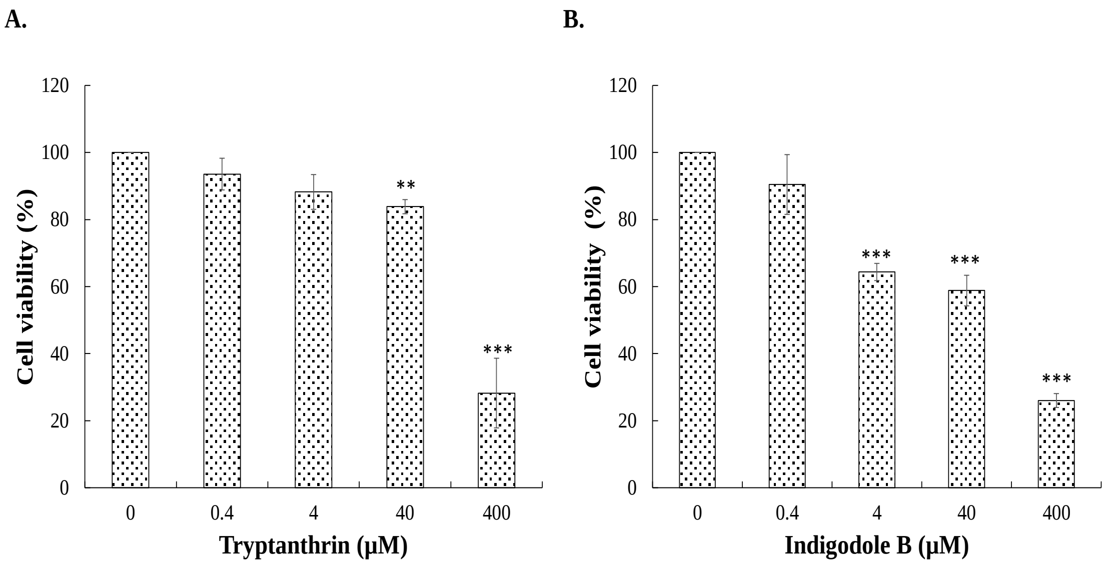

Supplemental Fig. 4. **Cell viability of Calu-3 cells treated with tryptanthrin (A) and indigodole B (5a*R*-ethyltryptanthrin) (B).** Cell viability of treated Calu-3 cells was evaluated using the MTT assay and calculated as the ratio of $OD_{570\text{–}630\ nm}$ of treated cells to $OD_{570\text{–}630\ nm}$ of untreated cells.
